# Synthesis, Characterization, and Biotoxicity of $\overset{⌢}{\text{N N}}$ Donor Sulphonamide Imine Silicon(IV) Complexes

**DOI:** 10.1155/BCA/2006/13743

**Published:** 2006-02-16

**Authors:** Mukta Jain, R. V. Singh

**Affiliations:** ^1^Department of Chemistry, Seth G. B. Podar College, Nawalgarh, Jhunjhunu, Rajasthan-333042, India; ^2^Department of Chemistry, University of Rajasthan, Jaipur-302004, India

## Abstract

The organosilicon derivatives of 2-[1-(2-furayl)ethyledene]sulphathiazole with organosilicon chlorides have been synthesised and characterized on the basis of analytical, conductance, and spectroscopic techniques. Probable trigonal bipyramidal and octahedral structures for the resulting derivatives have been proposed on the basis of electronic, IR, ^1^H, ^13^C NMR, and ^29^Si NMR spectral studies. In the search for better fungicides, bactericides, nematicides, and insecticides studies were conducted to assess the growth-inhibiting potential of the synthesized complexes against various pathogenic fungal, bacterial strains, root-knot nematode *Meloidogyne incognita*, and insect *Trogoderma granarium*. These studies demonstrate that the concentrations reached levels which are sufficient to inhibit and kill the pathogens, nematode, and insect.

## INTRODUCTION

Sulpha drugs are a group of compounds used for eliminating a wide range of infections in human and other animal systems. Many chemotherapeutically important sulpha drugs, like sulphadiazine, sulphathiazole, sulphamerazine, and so forth, possess SO_2_NH moiety which is an important toxophoric function [1]. The heterocyclic compounds with both sulphur and nitrogen atoms in the ring system have also been used in the synthesis of biologically active complexes. It is however noteworthy that the biological activity gets enhanced on undergoing complexation with metal ions [2]. Schiff bases and their metal complexes have exhibited biological activity as antibiotics, antiviral, and antitumor agents because of their specific structures. Heteronuclear Schiff base complexes have been found in applications as magnetic materials, catalysts and in the biological engineering field [3–6].

Organosilicon compounds of sulphur-containing ligands have attracted much attention recently due to their biological importance. The sulphur containing ligands are well known for their anticarcinogenic, antibacterial, tuberculostatic, antifungal, insecticidal, and acaricidal activities. It has been reported that the activity of sulphur-containing ligand increases on complexation [7–15]. The interest in organosilicon(IV) compounds is due to their versatile applicability in the pharmaceutical industries. Generally, organosilicon compounds seem to own their antitumour properties to the immuno defensive system of the organism [16–19]. The medical applications and effectiveness of the silatranes in the treatment of wounds and tumours are thought to be related to the role of silicon in the growth of epithelial and connective tissues and hair, where its function is to impart strengths, elasticity, and impermeability to water [20].

**Scheme 1:**
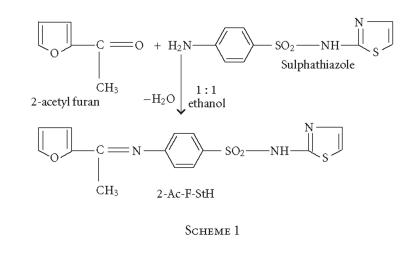


The preparation and characterization of one biologically active sulphonamide imine derived from 2-acetylfuran with sulphathiazole and its silicon(IV) complexes form the subject of this paper. The results of these investigations seem to be promising. Based on the coordination sites available in the ligand system, this has been classified as monobasic bidentate ligand (Scheme 1).

## EXPERIMENT

Adequate care was taken to keep the organosilicon(IV) complexes, chemicals, and glass apparatus free from moisture; clean and well-dried glass apparatus fitted with quickfit interchangeable standard ground joints was used throughout the experimental work. All the chemicals and solvents used were dried and purified by standard methods.

### Physical measurements and analytical methods

Nitrogen and sulfur were estimated by the Kjeldhal's and Messenger's methods, respectively. Silicon was determined gravimetrically as SiO_2_. Molecular weights were determined by the Rast camphor method (freezing point depression method) using resublimed camphor (MP 178°C).

### Conductance measurements

The conductance measurements were carried out in dry dimethylformamide (DMF) at room temperature using a systronics conductivity bridge (model 305) in conjunction with a cell having a cell constant of 1.0.

### Electronic spectra

The electronic spectra were recorded on a Perkin Elmer UV visible spectrophotometer in the range 200–600 nm, using dry methanol as the solvent.

### IR spectra

Infrared spectra were recorded on a Nicolet Magna FT-IR 550 spectrophotometer in KBr pellets.

### Nuclear magnetic resonance measurements

Multinuclear magnetic resonance spectra (^1^H, ^13^C, and ^29^Si) were recorded on an FX 90 Q JEOL spectrometer operating at 90 MHz.

### ^1^H NMR spectra

^1^H NMR spectra were recorded in deuterated methanol at 89.55 MHz using tetramethylsilane (TMS) as an internal standard.

### ^13^C NMR spectra

^13^C NMR spectra were recorded in dry methanol using TMS as the internal standard at 22.49 MHz.

### ^29^Si NMR spectra

^29^Si NMR spectra were recorded at 17.75 MHz using deuterated dimethylsulphoxide (DMSO-d_6_) as the solvent.

### Preparation of the ligand

The sulphonamide imine was prepared by the condensation of 2-acetylfuran with sulphathiazole in equimolar ratio in absolute alcohol. The contents were refluxed for 3–4 hours and the solid which separated out was filtered off, recrystallized from the same solvent (ethanol), and dried in vacuo. The physical properties and microanalysis of this sulphonamide imine are recorded in Table 1.

### Synthesis of the organosilicon(IV) complexes

For the synthesis of the complexes, first the sodium salt of the ligand was prepared by dissolving sodium metal (0.04–0.07 g) in 30 mL of methanol. Now to the weighed amount of organosilicon chlorides in 1 : 1 (0.38–0.51 g) or 1 : 2 molar ratios (0.11–0.29) in 20 mL methanol, the above prepared sodium salt of the ligand was added. The solution was refluxed for a period of 15–17 hours. The white precipitate of sodium chloride, formed during the course of the reaction, was removed by filtration and the filtrate was dried under reduced pressure. The resulting product was repeatedly washed with a mixture of methanol and *n*-hexane (1 : 1 v/v) and then finally dried for 3–4 hours. The purity was further checked by TLC using silica gel G. The details of these reactions and the analyses of the resulting products are recorded in Table 1.

## RESULTS AND DISCUSSION

**Scheme 2:**
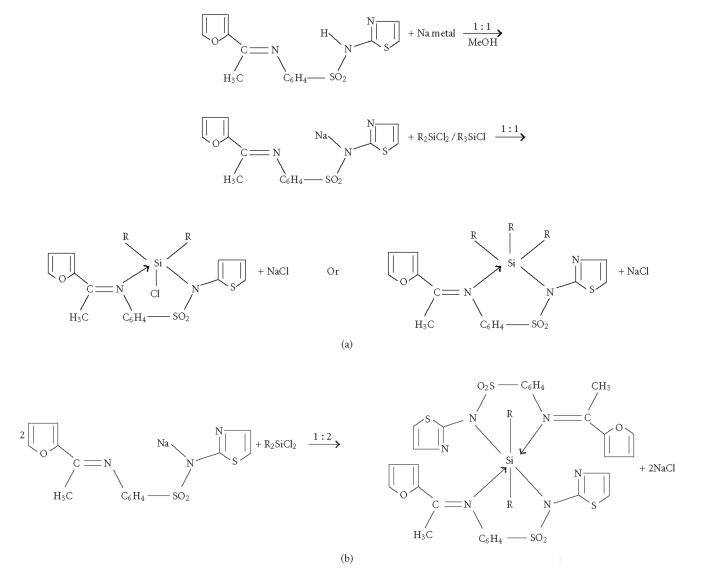
General equations showing the formations of the sodium salt and the complexes (R = Me and Ph).

The 1 : 1 and/or 1 : 2 molar reactions of Me_2_SiCl_2_, Ph_2_SiCl_2_, and Ph_3_SiCl with sulphonamide imine have led to the formation of Me_2_SiCl(2-Ac-F-St), Me_2_Si(2-Ac-F-St)_2_, Ph_2_SiCl(2-Ac-F-St), Ph_2_Si(2-Ac-F-St)_2_, and Ph_3_Si(2-Ac-F-St) types of complexes. The reactions have been carried out in perfectly dry methanolic medium and proceed smoothly with the precipitation of NaCl. These reactions can be represented by the general equations in Scheme 2 showing the formations of the sodium salt and the complexes.

The resulting coloured solids are soluble in most of the common organic solvents. These have been found to be monomeric as evidenced by their molecular weight determinations. The low values of molar conductivity (10–27 ohm^−1^ cm^2^ mol^−1^) of the resulting silicon complexes in anhydrous DMF show them to be nonelectrolytes in nature.

### UV spectra

The electronic spectra of the sulphonamide imine and its 1 : 1 and 1 : 2 organosilicon(IV) complexes have been recorded. The spectrum of the ligand shows a broad band at 370 nm which can be assigned to the n-π* transitions of the azomethine group. This band shows a blue shift in the silicon complexes appearing at 351, 353, 359, and 355, 362 nm for 1 : 1 and 1 : 2 derivatives, respectively, due to the polarisation within the >C=N chromophore caused due to formation of covalent silicon–nitrogen bond. The bands at 255 and 285 nm are due to *π*–π* transitions, within the benzene ring and (>C=N) band of the azomethine group, respectively. The K band *π*–π* showed a red shift due to the overlap of the central metal d-orbital with the p-orbital of the donor atom which causes an increase in conjugation and the B-bands undergo a hypsochromic shift in the complexes [21], see Table 2.

### IR spectra

The assignments of characteristic IR frequencies for the resulting complexes may be discussed as follows.

The IR spectra of these derivatives do not show any band in the region 3400-3150 cm^−1^ which could be assigned to *ν* NH. This clearly indicates the deprotonation of the ligand as a result of complexation with the silicon atom. A sharp band at 1628 cm^−1^ due to *ν*(>C=N) frequency of the free azomethine group in the ligand shifts to the lower frequency (ca 15 cm^−1^) in the silicon complexes and indicating thereby the coordination of the azomethine nitrogen to the silicon atom. A shift of this frequency to the higher and lower wave number side as well as the “no change” has also been reported in the literature [16].

In dimethylsilicon(IV) complexes, a band at ca 1420 cm^−1^ has been ascribed to the asymmetric deformation vibrations of (CH_3_−Si) group, whereas the band at ca 1270 cm^−1^ has been ascribed to the symmetric deformation mode of (CH_3_−Si) group. New bands are observed in the spectra of the complexes at ca 570–582 cm^−1^ due to the *ν*(Si ← N) vibrations. These remain absent in the spectrum of the ligand. A band due to *ν*(Si−Cl) at ca 423 and 439 cm^−1^ is observed in 1 : 1 diorganosilicon(IV) derivatives. It has been reported [16] that the cis form of such complexes gives rise to two *ν*(Si ← N ) bands, whereas in the transform only one IR active *ν*(Si ← N) band is observed. The presence of only one *ν*(Si ← N) band in the present case suggests that the complexes exist in the transform, see Table 3.

### ^1^H NMR spectra

The proton magnetic resonance spectral data of sulphonamide imine and its corresponding silicon complexes have been recorded in DMSO-d_6_. The chemical shift values relative to the TMS peak are listed in Table 4.

The broad signal due to the −NH proton in the ligand disappears in the case of silicon complexes showing the coordination of silicon to nitrogen after the deprotonation of the functional group. The azomethine proton signal due to methyl proton (${\text{H}}_{3}-\overset{\text{|}}{\text{C}}=\text{N}$) appears at δ 2.10 ppm in the ligand. The downfield shift of this position in the spectra of the complexes substantiates the coordination of azomethine nitrogen to the silicon atom. The additional signal in the region δ (1.01 and 1.13 ppm) in Me_2_SiCl(2-Ac-F-St) and Me_2_Si(2-Ac-F-St)_2_ types of complexes are due to Me_2_Si group.

The ligand shows a complex pattern in the region δ 8.10–6.92 ppm for the aromatic protons and this is observed in the region δ 8.78–6.95 ppm in the spectra of the organosilicon(IV) complexes. This shifting also supports the coordination through the nitrogen atom.

### ^13^C NMR spectra

The conclusions drawn from the UV, IR, and ^1^H NMR spectra are concurrent with the ^13^C NMR spectral data regarding the confirmation of the proposed structure. ^13^C NMR spectra of the ligand and its silicon complexes were also recorded in dry DMSO. The shifting of the signals due to carbon attached to the azomethine nitrogen in the spectra of the complexes further supports the involvement of this group in complexation [15]. Data are recorded in Table 5.

### ^29^Si NMR spectra

The ^29^Si NMR spectra of Me_2_SiCl(2-Ac-F-St), Ph_2_SiCl(2-Ac-F-St), and Ph_3_Si(2-Ac-F-St) give sharp signals at δ-91 to δ-98 ppm and the spectra of Me_2_Si(2-Ac-F-St)_2_ and Ph_2_Si(2-Ac-F-St)_2_ give sharp signals at δ-128 to δ-110 ppm, which clearly indicates the penta- and hexa-coordinated environment, respectively, around the silicon atom. Though, the exact geometries of these complexes can be suggested on the basis of X-ray crystal structure; inspite of our best efforts we could not develop a suitable crystal for the X-ray studies. Hence, X-ray data could not be included in the present paper.

**Figure 1:**
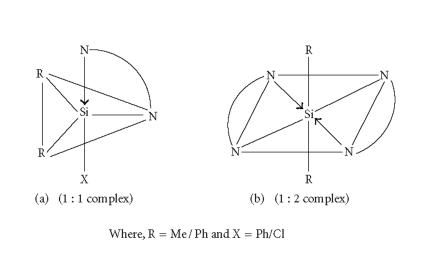
(a) The penta-coordinated trigonal bipyramidal and (b) hexa-coordinated octahedral geometries R = Me/Ph and X = Ph/Cl.

Thus, on the basis of the above spectral features, as well as the analytical data, the penta-coordinated trigonal bipyramidal and hexa-coordinated octahedral geometries shown in Figure 1 have been suggested for the organosilicon(IV) complexes.

## BIOLOGICAL ASPECTS

Fungicidal, bactericidal, nematicidal, and insecticidal activities of the sulphonamide imine and its respective organosilicon(IV) complexes against pathogenic fungi, bacteria, root-knot nematode, and insect are recorded in Tables 6–12.

### Antifungal screening

Like plant cells, fungi also possess cell walls but they cannot perform photosynthesis, moulds spoil food, damage potato, and crop plants (corn and wheat). They also cause rotting of clothes, shoes, and wooden materials. Some fungi cause diseases like athlete's foot and ring worm.

### Method

The antifungal activities were evaluated against *Macrophomina phaseolina, Aspergillus niger, Fusarium oxysporum*, and *Alternaria alternata* by agar plate technique [22]. The compounds were dissolved in 25, 50, and 100 ppm concentrations in methanol and then mixed with the medium. The linear growth of the fungus was obtained by measuring the diameter of the colony after 96 hours. The inhibition percentage was calculated as 100 (*D_fc_* − *D_ft_*)/*D_fc_*, where *D_fc_* and *D_ft_* are the diameters of the fungus colony in the control and the test plates, respectively.

### Antibacterial screening

Of all the microorganisms, bacteria are the most abundant. They generally reproduce quite fast, such as *P cepacicola* which reproduces itself every 9.5 minutes. However, some bacteria are very slow growing, such as those that cause tuberculosis and leprosy. This makes early diagnosis of these diseases rather difficult. The most common bacteria used for scientific research is *E coli*. Its normal living place is the lower human intestine (COLON).

### Method

Bactericidal activities were evaluated by the paper disc plate method [23]. The nutrient agar medium (peptone, beef extract, NaCl, and agar-agar) and 5 mm diameter paper discs (Whatman No. 1) were used. The compounds were dissolved in methanol in 500 and 1000 ppm concentrations. The filter paper discs were soaked in different solutions of the compounds, dried, and then placed in the petri plates previously seeded with the test organisms (*P cepacicola, E coli, K aerogenous*, and* S aureus*). The plates were incubated for 24–30 hours at 28±2°C and the inhibition zone around each disc was measured.

### Observations

The free ligand and its respective metal chelates were screened against selected fungi and bacteria to assess their potential as antimicrobial agents. The results are quite promising. The antimicrobial data reveal that the complexes are superior than the free ligands. The enhanced activity of the silicon chelates may be ascribed to the increased lipophilic nature of these complexes arising due to the chelation [24]. The toxicity increased as the concentration was increased. Further, the results of bioactivity were compared with the conventional fungicide, *Bavistin*, and the conventional bactericide, *Streptomycin*, taken as standards in either case.

In fungicide activity, most of the organosilicon(IV) complexes were able to inhibit and kill the pathogens at 50 ppm concentration, whilst 100 ppm concentration proved invariably fatal. None of the fungi was able to withstand this concentration. In bactericidal activity, the complexes exhibited remarkable potential in inhibiting the growth of pathogens. Many of the complexes were found to be even more toxic than the standard. Thus, it can be postulated that further intensive studies of these complexes in this direction as well as in agriculture could lead to the interesting results.

### Nematicidal activity

Development of the concept of pest management and their implementation have led to a greater appreciation of the need for a wide range of tactics for nematode control. The objective of nematode control is to improve growth and yield of plants, which can be achieved through a reduction of the nematode population in soil or in plants, or through a reduction of their damage. Chemical method can be used to control nematodes [25]. *M incognita* produce galls on the roots of many host plants and responsible for 44.87 percent of yield loss in brinjal [26].

### Method

First of all we applied different concentrations (25, 50, and 100) in ppm of complexes and ligand on root-knot nematode *M incognita spp.* in a step-by-step [27] procedure. For experiment, egg masses were separated from heavily infected brinjal roots and washed under running water. After cutting the roots, one percent of sodium hypochlorite solution was added, shaked, and then sieved through 150 and 400 sieves. Then the eggs of nematode were counted and replicated three times. At this experiment, temperature range was 30 ± 2°C.

### Observations

Maximum hatching was recorded in control. All the metal complexes are more toxic than the ligand and all bimolar complexes are more active than unimolar organosilicon derivatives. Dimethylsilicon(IV) complexes are less hazardous than diphenylsilicon(IV) complexes. The activity increases with increasing the concentration of the solutions.

### Mode of action [15]

Much smaller amounts of the nonfumigant and fumigant nematicides are needed in plant protection against nematode because the indirect hematostatic effects of non-fumigant nematicides resulting from impairment of neuromuscular activity, interfere with movement, feeding, invasion, development, reproduction, fecundity, and hatching of nematodes which are considered more important than their direct killing action.

### Insecticidal activity

Many insects cause injury to economic plants by feeding on them externally: by chewing their leaves or other part: In order to raise more food, man has devised methods to alter normal population growth of many insect pests by reducing their chance for survival. To control the insect pests, the man since long has been employing various strategies which include mechanical, physical, chemical, and biological methods [28].

### Methods

#### Ovicidal

To determine the efficacy of complexes as ovicide, eggs were treated by contact method. By spreading 1 mL of complex solutions on petri dishes (5.0 cm diameter), a thin film of 100 and 200 concentrations were prepared. The solvent was allowed to evaporate 200 eggs for 0–24 hours and kept in contact with the insecticidal film through out their incubation period. A control with each experiment was also run in which the eggs were kept in 1 mL of solvent. By Abott's formula [29], percentage of egg of mortality and percentage of corrected egg of mortality were calculated.

(1) $$\text{\% corrected mortality = }\frac{K_{T}\;-\;K_{C}}{100\;-\;K_{C}}\;\times \;100$$,

where *K_T_* = % kill in treated, *K_C_* = % kill in control.

#### Larvicidal

By feeding method larvicidal efficacy of the synthesized chemicals was assessed. The last instar larvae were separated from subculture and kept in vials containing 5 g of topically treated wheat grains with 1 mL of chemicals. Until the pupal formation, larvae were allowed to continue their development on this diet, replicated thrice, each dose. The food was treated with solvent only in control. By Abott's formula, larval of mortality and percentage of corrected of mortality were calculated.

#### Pupicidal

From the subculture, the last larval instars were stored out and were kept in separate container. Pupal of known age (0–12 hours) were taken out and were dipped in the desired concentration (100 and 200) of the chemicals along with a control of three replicates that were set for each dose and total emergence and pupal of mortality were recorded after 96 hours. By Abott's formula, percentage of pupal of mortality and percentage of pupal corrected of mortality were calculated.

#### Adulticidal

By contact method the adulticidal action was assessed. With 1 mL of respective doses, 5 g of wheat grains were treated. The solvent was allowed to evaporate completely. Along with a control, experiment was replicated thrice. Newly emerged adults were taken from the subculture and were released in the plastic vials containing treated food. After 48-hour observations were taken and by Abott's formula, percentage of corrected of mortality was calculated.

### Mode of action [30]

Some insecticides are physical poisons causing asphyxiation, some are protoplasmic poisons, a few are respiratory poisons, but the majority of them are nerve poisons. The action of insecticides upsets the normal behaviour and actions of the target organisms.

Ovicidal, larvicidal, pupicidal, and adulticidal results are shown in Tables 9, 10, 11, and 12. The data indicated the same observations as were observed in nematicidal activity.

## Figures and Tables

**Table 1:** Analysis and physical properties of the ligand and its silicon complexes.

Compound							Elemental analysis (%)						
						
	Reactant (g)			Colour	Yield	MP	C	H	N	S	Si	Cl	Mol Wt
	M*	LH*	Na*	and state	(%)	(°C)	Found	Found	Found	Found	Found	Found	Found
							(Calcd)	(Calcd)	(Calcd)	(Calcd)	(Calcd)	(Calcd)	(Calcd)

(2-Ac-F-StH)	—	—	—	Light yellow	73	124–130	51.62	3.51	11.84	18.19	—	—	325
							(51.86)	(3.77)	(12.09)	(18.45)			(347.39)
Me_2_SiCl(2-Ac-F-St)	0.38	1.02	0.07	Dark brown	74	71–73	46.18	3.88	9.26	14.19	6.07	8.00	412
				solid			(46.40)	(4.12)	(9.55)	(14.57)	(6.38)	(8.05)	(439.99)
Me_2_Si(2-Ac-F-St)_2_	0.11	0.61	0.04	Light brown	76	109–111	50.99	3.67	10.77	16.70	3.48	—	738
				solid			(51.18)	(4.02)	(11.19)	(17.07)	(3.74)		(750.92)
Ph_2_SiCl(2-Ac-F-St)	0.48	0.66	0.04	Dark brown	74	149–151	57.15	3.58	7.09	11.00	4.71	5.92	542
				solid			(57.48)	(3.93)	(7.44)	(11.36)	(4.97)	(6.28)	(564.13)
Ph_2_Si(2-Ac-F-St)_2_	0.29	0.81	0.05	Dark brown	77	155–157	57.31	3.74	9.42	14.19	3.00	—	858
				solid			(57.64)	(3.91)	(9.60)	(14.65)	(3.20)		(875.05)
Ph_3_Si(2-Ac-F-St)	0.51	0.60	0.04	Brown solid	81	90–92	65.02	4.12	6.68	10.19	4.22	—	588
							(65.42)	(4.49)	(6.93)	(10.58)	(4.63)		(605.78)

*M = silicon compound, LH = ligand, and Na = sodium metal.

**Table 2:** UV spectral data of the ligand and its silicon complexes.

Ligand/complex	*n*-π* (nm)	*π*-π* (nm)	*π*-π* (nm)
	>C=N	C_6_H_5_ ring	>C=N

(2-Ac-F-StH)	370	255	285
Me_2_SiCl(2-Ac-F-St)	359	273	281
Me_2_Si(2-Ac-F-St)_2_	362	276	278
Ph_2_SiCl(2-Ac-F-St)	351	280	275
Ph_2_Si(2-Ac-F-St)_2_	355	285	271
Ph_3_Si(2-Ac-F-St)	353	290	268

**Table 3:** IR spectral data (cm^−1^) of the ligand and its silicon complexes.

Compound/ligand	*ν*(NH)	*ν*(C=N)	*ν*(Si ← N)	*ν*(Si−Cl)

(2-Ac-F-StH)	3400-3150 (m)*	1628 (vs)*	—	—
Me_2_SiCl(2-Ac-F-St)	—	1622	577 w*	423 m
Me_2_Si(2-Ac-F-St)_2_	—	1625	582 w	—
Ph_2_SiCl(2-Ac-F-St)	—	1619	574 w	439 m
Ph_2_Si(2-Ac-F-St)_2_	—	1613	576 w	—
Ph_3_Si(2-Ac-F-St)	—	1616	570 w	—

*m = medium, vs = very strong, and w = weak.

**Table 4:** ^1^H NMR spectral data (δ, ppm) of the ligand and its silicon complexes.

Ligand/complex	Si−CH_3_	CH_3_	NH	Aromatic proton	^29^Si NMR

(2-Ac-F-StH)	—	2.10 (3H, s*)	10.54 (br*, 1H)	8.10-6.92 (m)*	—
Me_2_SiCl(2-Ac-F-St)	1.01 (1s, 6H)	2.25 (3H, s)	—	8.36-7.20 (m)	−98 (ppm)
Me_2_Si(2-Ac-F-St)_2_	1.13 (s, 6H)	2.17 (6H, s)	—	8.784-7.00 (m)	−128 (ppm)
Ph_2_SiCl(2-Ac-F-St)	—	2.22 (3H, s)	—	8.48-6.95 (m)	−94 (ppm)
Ph_2_Si(2-Ac-F-St)_2_	—	2.15 (6H, s)	—	8.56-7.30 (m)	−110 (ppm)
Ph_3_Si(2-Ac-F-St)	—	2.19 (3H, s)	—	8.51-7.14 (m)	−91 (ppm)

*m = multiplet, br = broad, and s = singlet.

**Table 5:**
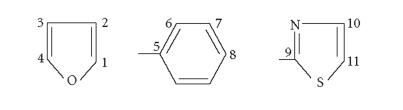
^13^C NMR spectral data (δ, ppm) of the ligand and its silicon complexes.

Ligand/complex	Azomethine C-atom	Si−CH_3_	C_1_	C_2_	C_3_	C_4_	C_9_	C_11_
			C_5_	C_6_	C_7_	C_8_	C_10_	

(2-Ac-F-StH)	155.91	—	146.02	138.99	120.98	143.94	152.60	151.80
			128.01	122.46	124.01	125.98	150.00	
Me_2_SiCl(2-Ac-F-St)	144.76	13.98	148.91	139.21	121.12	142.92	149.20	150.70
			125.96	120.24	123.67	119.76	150.95	
Me_2_Si(2-Ac-F-St)_2_	148.51	15.01	147.69	140.96	121.32	143.01	149.45	149.85
			124.21	120.96	122.21	119.10	151.00	
Ph_2_SiCl(2-Ac-F-St)	153.46	—	146.36	137.01	120.76	142.10	151.20	149.70
			126.01	121.78	119.98	122.46	150.15	
Ph_2_Si(2-Ac-F-St)_2_	146.76	—	145.16	138.06	127.92	143.21	147.20	150.12
			128.96	121.02	120.21	123.74	149.80	
Ph_3_Si(2-Ac-F-St)	154.90	—	144.05	133.42	120.81	143.40	148.78	149.40
			127.01	121.98	123.32	124.86	150.55	

**Table 6:** Fungicidal screening data of the ligand and its silicon complexes inhibition percentage after 96 hours and SD values (25, 50, and 100 are concentrations in ppm).

Ligand/complex	*Aspergillus niger*			*Macrophomina phaseolina*			*Fusarium oxysporum*			*Alternaria alternata*		

	25	50	100	25	50	100	25	50	100	25	50	100

(2-Ac-F-StH)	34	53	61	35	50	68	39	56	65	43	60	66
	(50.72)	(38.37)	(37.75)	(51.38)	(39.02)	(29.16)	(44.28)	(38.46)	(35.00)	(39.44)	(30.23)	(34.00)
	
Me_2_SiCl(2-Ac-F-St)	37	56	72	38	52	71	42	59	71	45	62	68
	(46.37)	(34.88)	(36.11)	(47.22)	(36.58)	(26.04)	(40.00)	(35.16)	(29.00)	(36.62)	(27.91)	(32)
	
Me_2_Si(2-Ac-F-St)_2_	42	63	78	41	57	74	46	65	74	47	65	72
	(39.13)	(26.74)	(20.40)	(43.05)	(30.48)	(22.44)	(35.28)	(28.57)	(26.00)	(33.80)	(24.42)	(28.00)
	
Ph_2_SiCl(2-Ac-F-St)	38	57	76	40	53	72	43	61	73	46	63	70
	(44.92)	(33.72)	(22.44)	(44.44)	(35.36)	(26.53)	(38.57)	(32.96)	(27.00)	(35.21)	(26.74)	(30.00)
	
Ph_2_Si(2-Ac-F-St)_2_	44	66	82	47	61	80	48	67	78	49	66	76
	(36.23)	(23.25)	(16.32)	(34.72)	(25.60)	(18.36)	(31.42)	(26.37)	(22.00)	(30.99)	(23.26)	(24.00)
	
Ph_3_Si(2-Ac-F-St)	40	60	80	42	54	73	45	63	75	47	64	71
	(42.02)	(30.23)	(18.36)	(41.66)	(34.14)	(25.51)	(35.71)	(30.76)	(25.00)	(33.80)	(25.58)	(29.00)
	
Bavistin	69	86	98	72	82	96	70	91	100	71	86	100

**Table 7:** Bactericidal screening data of the ligand and its silicon complexes diameter inhibition zone (mm) after 24 hours (500 and 1000 are concentrations in ppm).

Ligand/complex	*Esherichia coli* (−)		*Klebsiella aerogenous* (−)		*Pseudomonas cepacicola* (−)		*Staphylococcus aureus* (+)	

	500	1000	500	1000	500	1000	500	1000

(2-Ac-F-StH)	6	6	6	11	10	12	9	13
Me_2_SiCl(2-Ac-F-St)	8	12	9	15	12	14	12	14
Me_2_Si(2-Ac-F-St)_2_	10	16	11	17	15	17	16	18
Ph_2_SiCl(2-Ac-F-St)	10	14	10	16	14	16	15	16
Ph_2_Si(2-Ac-F-St)_2_	13	18	14	19	17	19	18	19
Ph_3_Si(2-Ac-F-St)	11	16	12	17	15	17	16	17
Streptomycin	1	2	3	5	2	5	15	17

**Table 8:** Nematicidal screening data of the ligand and its silicon complexes (25, 50, and 100 are concentrations in ppm).

Ligand/complex	(% of hatching *M incognita*)		

	25	50	100

(2-Ac-F-StH)	22.5	19.0	15.0
Me_2_SiCl(2-Ac-F-St)	20.2	16.5	No hatching
Me_2_Si(2-Ac-F-St)_2_	18.5	14.9	No hatching
Ph_2_SiCl(2-Ac-F-St)	19.6	16.4	No hatching
Ph_2_Si(2-Ac-F-St)_2_	15.9	11.9	No hatching
Ph_3_Si(2-Ac-F-St)	18.6	14.0	No hatching

**Table 9:** Ovicidal screening data of the ligand and its silicon complexes (100 and 200 are concentrations in ppm).

Ligand/complex	Dose level	Average no. of	Average no. of	% eggs	% eggs	% corrected
		eggs hatched	eggs unhatched	hatching	unhatched	mortality

(2-Ac-F-StH)	100	15	5	75	25	21.05
	200	11	9	55	45	42.10
Me_2_SiCl(2-Ac-F-St)	100	13	7	65	35	31.57
	200	9	11	45	55	52.63
Me_2_Si(2-Ac-F-St)_2_	100	9	11	45	55	52.63
	200	7	13	35	65	63.15
Ph_2_SiCl(2-Ac-F-St)	100	11	9	55	45	42.10
	200	7	13	35	65	63.15
Ph_2_Si(2-Ac-F-St)_2_	100	8	12	40	60	57.89
	200	5	15	25	75	73.68
Ph_3_Si(2-Ac-F-St)	100	10	10	50	50	47.36
	200	7	13	35	65	63.15
Control	—	19	1	95	5	—

**Table 10:** Larvicidal screening data of the ligand and its silicon complexes (100 and 200 are concentrations in ppm).

Ligand/complex	Dose level	Average no. of	Average no. of	% pupal	% larval	% corrected
		pupal formed	dead larvae	formation	mortality	mortality

(2-Ac-F-StH)	100	16	4	80	20	15.78
	200	13	7	65	35	31.57
Me_2_SiCl(2-Ac-F-St)	100	13	7	65	35	31.57
	200	10	10	50	50	47.36
Me_2_Si(2-Ac-F-St)_2_	100	9	11	45	55	52.63
	200	7	13	35	65	63.15
Ph_2_SiCl(2-Ac-F-St)	100	11	9	55	45	42.10
	200	8	12	40	60	57.89
Ph_2_Si(2-Ac-F-St)_2_	100	7	13	35	65	63.15
	200	5	15	25	75	73.68
Ph_3_Si(2-Ac-F-St)	100	11	9	55	45	42.10
	200	6	14	30	70	68.42
Control	—	19	1	95	5	—

**Table 11:** Pupicidal screening data of the ligand and its silicon complexes (100 and 200 are concentrations in ppm).

Ligand/complex	Dose level	Average no. of	Average no. of	% emerged	% pupal	% corrected
		adults emerged	pupal mortality	adult	mortality	mortality

(2-Ac-F-StH)	100	15	5	75	25	21.05
	200	14	6	70	30	26.31
Me_2_SiCl(2-Ac-F-St)	100	14	6	70	30	26.31
	200	11	9	55	45	42.10
Me_2_Si(2-Ac-F-St)_2_	100	10	10	50	50	47.36
	200	6	14	30	70	68.42
Ph_2_SiCl(2-Ac-F-St)	100	12	8	60	40	38.84
	200	8	12	40	60	57.89
Ph_2_Si(2-Ac-F-St)_2_	100	9	11	45	55	52.63
	200	5	15	25	75	73.68
Ph_3_Si(2-Ac-F-St)	100	11	9	55	45	42.10
	200	7	13	35	65	63.15
Control	—	19	1	95	5	—

**Table 12:** Adulticidal screening data of the ligand and its silicon complexes (100 and 200 are concentrations in ppm).

Ligand/complex	Dose level	Average no. of	Average mortality	% adult	% corrected
		adults in each vial	after 48 hours	mortality	mortality

(2-Ac-F-StH)	100	20	4	20	15.78
	200	20	6	30	26.31
Me_2_SiCl(2-Ac-F-St)	100	20	7	35	31.57
	200	20	11	55	52.63
Me_2_Si(2-Ac-F-St)_2_	100	20	11	55	52.63
	200	20	14	70	68.42
Ph_2_SiCl(2-Ac-F-St)	100	20	9	45	42.10
	200	20	12	60	57.10
Ph_2_Si(2-Ac-F-St)_2_	100	20	12	60	57.89
	200	20	15	75	57.89
Ph_3_Si(2-Ac-F-St)	100	20	10	50	73.68
	200	20	4	70	47.36
Control	—	20	1	5	68.42
